# The interferon-stimulated exosomal hACE2 potently inhibits SARS-CoV-2 replication through competitively blocking the virus entry

**DOI:** 10.1038/s41392-021-00604-5

**Published:** 2021-05-12

**Authors:** Junsong Zhang, Feng Huang, Baijin Xia, Yaochang Yuan, Fei Yu, Guanwen Wang, Qianyu Chen, Qian Wang, Yuzhuang Li, Rong Li, Zheng Song, Ting Pan, Jingliang Chen, Gen Lu, Hui Zhang

**Affiliations:** 1 Guangdong Provincial People’s Hospital, Guangdong Academy of Medical Sciences, Guangzhou, Guangdong China; 2 Institute of Human Virology, Key Laboratory of Tropical Disease Control of Ministry Education, Guangdong Engineering Research Center for Antimicrobial Agent and Immunotechnology, Zhongshan School of Medicine, Sun Yat-sen University, Guangzhou, Guangdong China; 3 Bioland Laboratory (Guangzhou Regenerative Medicine and Health Guangdong Laboratory), Guangzhou, Guangdong China; 4 School of Medicine, Sun Yat-sen University, Guangzhou, Guangdong China; 5 Infectious Disease Center, Guangzhou Eighth People’s Hospital, Guangzhou Medical University, Guangzhou, China; 6 Department of Respiratory Diseases, Guangzhou Women and Children Hospital, Guangzhou, Guangdong China

**Keywords:** Microbiology, Innate immunity

## Abstract

Since the outbreak of coronavirus disease 2019 (COVID-19), it has become a global pandemic. The spike (S) protein of etiologic severe acute respiratory syndrome coronavirus 2 (SARS-CoV-2) specifically recognizes human angiotensin-converting enzyme 2 (hACE2) as its receptor, which is recently identified as an interferon (IFN)-stimulated gene. Here, we find that hACE2 exists on the surface of exosomes released by different cell types, and the expression of exosomal hACE2 is increased by IFNα/β treatment. In particular, exosomal hACE2 can specifically block the cell entry of SARS-CoV-2, subsequently inhibit the replication of SARS-CoV-2 in vitro and ex vivo. Our findings have indicated that IFN is able to upregulate a viral receptor on the exosomes which competitively block the virus entry, exhibiting a potential antiviral strategy.

## Introduction

The emergence of severe acute respiratory syndrome (SARS)-like coronavirus (SARS-CoV-2) has caused a highly contagious infectious disease (COVID-19).^1–3^ Similar to individuals infected by pathogenic SARS-CoV in 2003 and MERS-CoV in 2012, the patients infected by SARS-CoV-2 showed a range of clinical symptoms, such as high fever and acute pneumonia with an estimated mortality rate ranging from 3 to 5%.^4–6^ Since the initial outbreak of COVID-19, the disease has spread to more than 180 countries and areas worldwide.^7^ The worldwide expansion of COVID-19 has continued to threaten global health and economy. Thus, it is urgent to develop novel anti-SARS-CoV-2 therapeutics and prophylactics for the treatment of COVID-19.

SARS-CoV-2 belongs to a lineage B beta-CoV and is closely linked to SARS-CoV based on phylogenetical analysis.^8,9^ SARS-CoV-2 infection is initiated by interaction of the viral spike (S) protein with the receptor on the cell surface. Similar with SARS-CoV, the S1 subunit of S protein of SARS-CoV-2 utilizes human angiotensin-converting enzyme 2 (hACE2) as the functional receptor to infect the target cells.^10–14^ The binding affinity of S protein of SARS-CoV-2 is even higher than that of SARS-CoV, which may contribute to higher infectivity and transmissibility of SARS-CoV-2 infection.^13^ Recombinant ACE2 (rACE2) has shown to protect the lung from injury,^15^ and human recombinant soluble ACE2 (hrsACE2) is currently reported to inhibit the infection of SARS-CoV-2 in Vero cells and in engineered human blood vessel organoids and human kidney organoids,^16^ implying that ACE2 plays a critical role in COVID-19 therapy.

During virus infection, type I interferon (IFN) is induced as the initial host defense against the viral invasion. Type I IFNs have broad-spectrum antiviral activities against viruses in a wide range of cell types and acting as a mediator of adaptive immune response.^17,18^ Humans produce 13 types of type I IFN including numerous IFNα subtypes and IFNβ, which ultimately induces the expression of a number of genes, so-called IFN-stimulated genes (ISGs).^19,20^ ISGs play critical roles in antiviral response, such that anti-HIV host factors APOBEC3G identified as an ISG by our group and others.^21–23^ As type I IFN is a very powerful defense maneuver for host cells to fight against viral infection, many viruses will evade the IFN-induced response by various strategies. Therefore, it is critical to identify ISGs and the strategies used by viruses to evade the response, and the exogenous type I IFN or molecules that mimic IFN responses will be of great potential for future antiviral immunotherapies.

Exosomes are membrane-bound extracellular vesicles (EVs) with a diameter 40–150 nm, which are released from cell into the extracellular space to influence the extracellular environment.^24,25^ During the maturation of endosomes, endocytic vesicles arise in lipid raft domains of the plasma membrane through endocytosis, leading to the intracellular formation of early endosomes, which become late endosomes with the assistance of the Golgi complex. These late endosomes further develop to multivesicular bodies (MVBs). MVBs could fuse with either lysosomes for degradation, fuse with the plasma membrane for the release of their internal vesicles, such as exosomes into the extracellular space, or incorporate into the plasma membrane.^26,27^ The endosomal sorting complex required for transport (ESCRT) family plays a crucial role in the biogenesis and cargo sorting of exosomes. Especially, ESCRT family members, such as Hrs, Tsg101, and Alix are involved in the regulation of exosmal biogenesis.^28^ The depletion of Hrs reduces the secretion of exosomes.^29,30^ Besides these ESCRT family members, exosomes also contain other conserved proteins, such as CD9, CD63, and CD81.^25,26^ Many studies have demonstrated that exosomes can play significant roles in the treatment of many diseases, including cancer, cardiovascular diseases, and pathogenic infections.^31–34^ Exosomes have the potential to serve as vaccine and drug carriers engineered by various methods. It seems that exosome-based strategies may be a safe and effective therapeutic tool for the treatment of COVID-19.

While analyzing the expression and mechanisms regulating levels of hACE2 in different cell lines, we found that hACE2 can be secreted by different cell lines including human bronchial epithelial (16HBE), H1299, and HEK293T cells besides express on their cell surface. The expression of exosomal hACE2 was downregulated after depletion of *Hrs* using Hrs-specific shRNAs, and upregulated by IFNα/β treatment. Exosomal hACE2 can interact with S protein on the surface of SARS-CoV-2-S/HIV-1 pseudovirions and purified receptor-binding domain (RBD) of S protein. Thus, we utilized the purified hACE2-exosome to inhibit SARS-CoV-2 infection and found that the cell entry of SARS-CoV-2-S/HIV-1 pseudovirions was blocked after co-cultured with hACE2-exosome or IFNα/β-treated exosome. Remarkably, the replication of wild-type SARS-CoV-2 was inhibited in vitro and ex vivo after co-cultured with hACE2-exosome. Our findings have unfolded the mechanisms regulating levels of exosomal hACE2 and may present a novel therapeutic strategy for COVID-19 therapy.

## Results

### hACE2 is specifically expressed on the surface of exosomes and regulated by IFNα/β

Type I IFN plays a critical role during the initial host defense against the viral invasion, and accumulating data have indicated that IFNα/β treatment can inhibit SARS-CoV-2 replication in vitro and in vivo.^35–39^ We also found that IFNα/β treatment significantly inhibited the replication of SARS-CoV-2 in cells (Supplementary Fig. 1a), implying the critical roles of type I IFNs in anti-SARS-CoV-2 treatment. Thus, when analyzed signaling pathway involved in regulating the expression of hACE2, we repeated the results reported that hACE2 is upregulated by IFN signal.^40^ Accordingly, we found that hACE2 was indeed stimulated by IFNα and IFNβ in HEK293T cells and human airway cell lines including 16HBE, and H1299 cells (Supplementary Fig. 1b, c). This enhancement is dose-dependent and showed as a time-course manner for both IFNα and IFNβ treatment (Supplementary Fig. 1d–g). This phenomenon seems illogical as IFN is well known for its antiviral activity and it is unlikely for IFN to assist the viral entrance by upregulating the expression of viral receptor on the cell surface. Nevertheless, it is reasonably assumed that the upregulated ACE2 could be secreted from the cells and competitively inhibited the interaction between hACE2 on the surface of target cells and the viral particles.

**Fig. 1:**
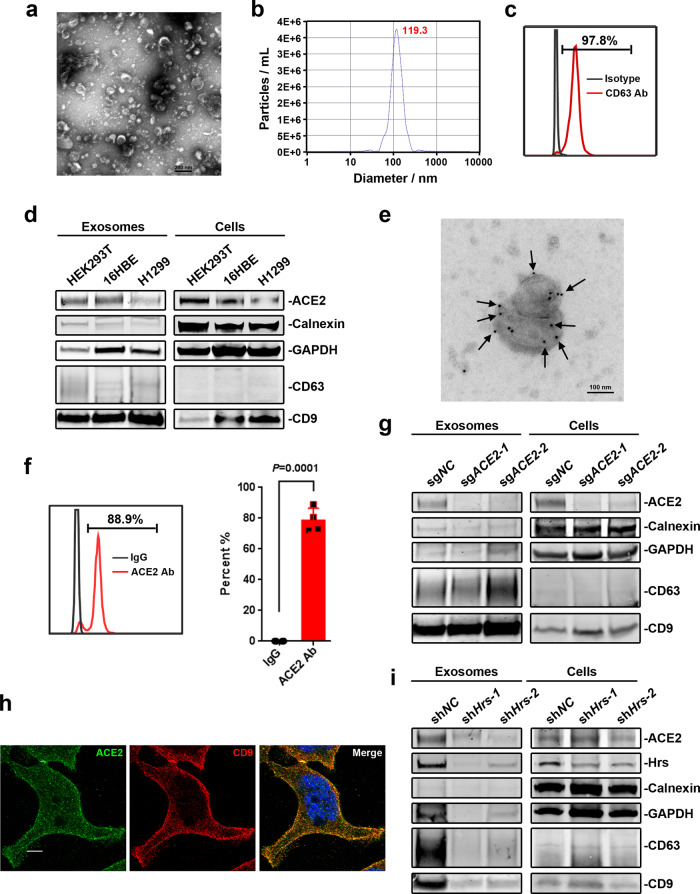
hACE2 is specifically expressed on the surface of exosomes. **a** A representative of TEM image of exosomes purified from HEK293T cells. Scale bar, 200 nm. **b** The size and concentration of purified exosomes derived from HEK293T cells by NTA. The size of 97.5% of total purified exosomes is about 119.3 nm. **c** The purified exosomes derived from HEK293T cells were further enriched with Human CD63 Isolation/Detection Reagent and stained with anti-CD63 antibody, followed by flow cytometry analysis. **d** Western blot analysis of the whole cell lysates (Cells) and purified exosomes (Exosomes) from HEK293T, 16HBE, and H1299 cells. **e** A representative TEM image of HEK293T cell-derived exosomes immunogold-labeled with anti-ACE2 antibodies. Two exosomes close together were shown in the picture. Arrowheads indicate 10 nm gold particles. Scale bar, 100 nm. **f** The purified exosomes derived from HEK293T cells were further enriched with Human CD63 Isolation/Detection Reagent and stained with anti-ACE2 antibody, followed by flow cytometry analysis (Left panel). Right: Frequency of ACE2^+^ exosomes in left. Data were shown as the mean ± SD of four independent experiments, and compared using the two-tailed student’s *t* test. ***P < 0.001. **g** Western blot analysis of the whole cell lysates and purified exosomes from sg*ACE2* knockdown cell lines or control cell line. **h** The confocal image of intracellular hACE2 and exosome marker CD9 in HEK293T. The cells were stained with anti-ACE2 (green) and anti-CD9 (red) antibodies. The nucleus was stained with DAPI (blue). Scale bars, 5 μm. **i** Western blot analysis of the whole cell lysates and purified exosomes from sh*Hrs* knockdown cell lines or control cell line

To explore whether hACE2 could express in the form of membrane vesicles secreted from cells, we purified exosomes from cell supernatant using differential ultracentrifugation, and verified the successful purification of exosomes by transmission electron microscopy (TEM), nanoparticle tracking analysis (NTA), and flow cytometry (Fig. 1a–c). Western blot, immune-electron microscopy, and flow cytometry analysis revealed that hACE2 existed on the surface of exosomes from different cell lines (Fig. 1d–f, Supplementary Fig. 2a–d). Of note, the exosomes derived from HEK293T cells could not be labeled with anti-TSG101 antibody (Supplementary Fig. 2e, left panel), while could be labeled with anti-CD63 and anti-ACE2 antibodies when using the same protocol. However, when we treated purified exosomes with 0.001% Triton X-100 for 5 min before antibody labeling,^41^ we found that exosomes derived from HEK293T cells could be labeled with anti-TSG101 antibody under this circumstance (Supplementary Fig. 2e, right panel). The expression of both cellular hACE2 and exosomal hACE2 was decreased after depletion of *hACE2* with hACE2-specific sgRNAs, further indicating that hACE2 specifically existed in exosomes (Fig. 1g and Supplementary Fig. 2b). hACE2 was also co-localized with the exosome marker CD9 in cells (Fig. 1h). In addition, when we depleted the expression of the ESCRT subunit *Hrs* with Hrs-specific shRNAs, we found a decrease in the level of exosomal hACE2 (Fig. 1i). Collectively, these results show that hACE2 is specifically expressed on the surface of exosomes.

**Fig. 2:**
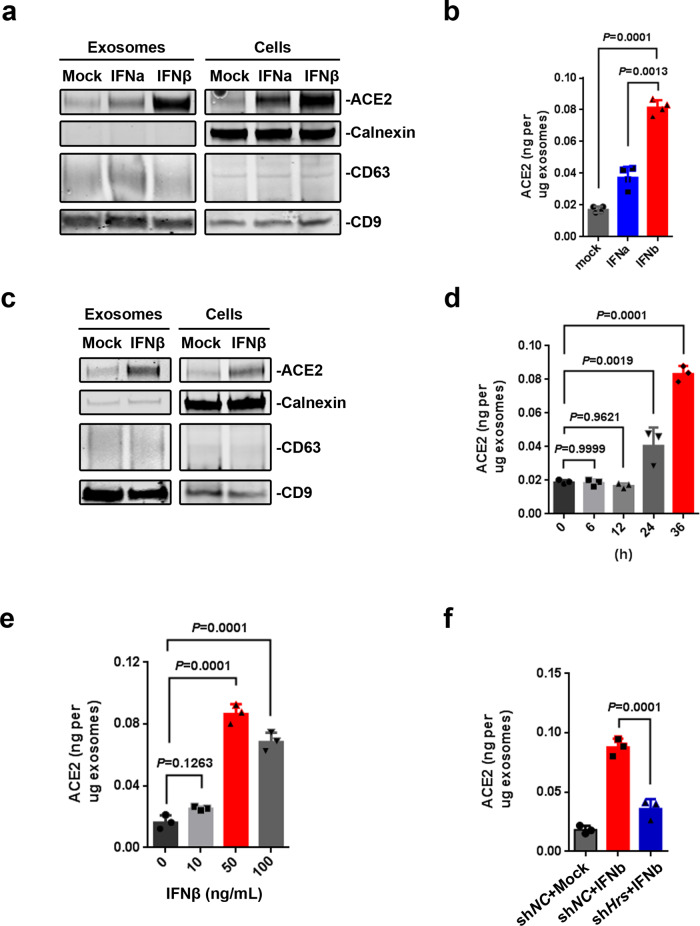
Exosomal hACE2 is stimulated by IFNα/β. **a** Western blot analysis of the whole cell lysates and purified exosomes from HEK293T cells treated with IFNα/β (50 ng/ml for 36 h) or not (Mock). **b** ELISA of hACE2 on exosomes derived from HEK293T cells treated with IFNα/β (50 ng/ml for 36 h) or not. **c** Western blot analysis of the whole cell lysates and purified exosomes from 16HBE cells treated with IFNβ (50 ng/ml for 48 h) or not (Mock). **d** ELISA of hACE2 on exosomes derived from HEK293T cells treated with 50 ng/ml of IFNβ for different time points. **e** ELISA of hACE2 on exosomes derived from HEK293T cells treated with different doses of IFNβ for 36 h. **f** ELISA of hACE2 on exosomes derived from Hrs knockdown cell line (sh*Hrs*-1 cell line) or control cell line treated with IFNβ (50 ng/ml for 36 h) or not. Data were shown as the mean ± SD of three independent experiments, and compared using one-way ANOVA test, in which each column compared to the first column of each graph. **P < 0.01; ***P < 0.001; ns not significant

Furthermore, treatment of cells with IFNα and IFNβ led to an increase in both the cellular and exosomal hACE2 from HEK293T cells (Fig. 2a, b and Supplementary Fig. 2f) and 16HBE cells (Fig. 2c and Supplementary Fig. 2g). IFNβ stimulated the expression of exosomal hACE2 in the time- and dose-dependent manners (Fig. 2d, e). The increase exosomal hACE2 induced by IFNβ was downregulated after the depletion of the expression of *Hrs* with Hrs-specific shRNAs (Fig. 2f), indicating that exosomal hACE2 is stimulated by IFNα/β.

### Exosomal hACE2 competitively blocks the cell entry of SARS-CoV-2

**Fig. 3:**
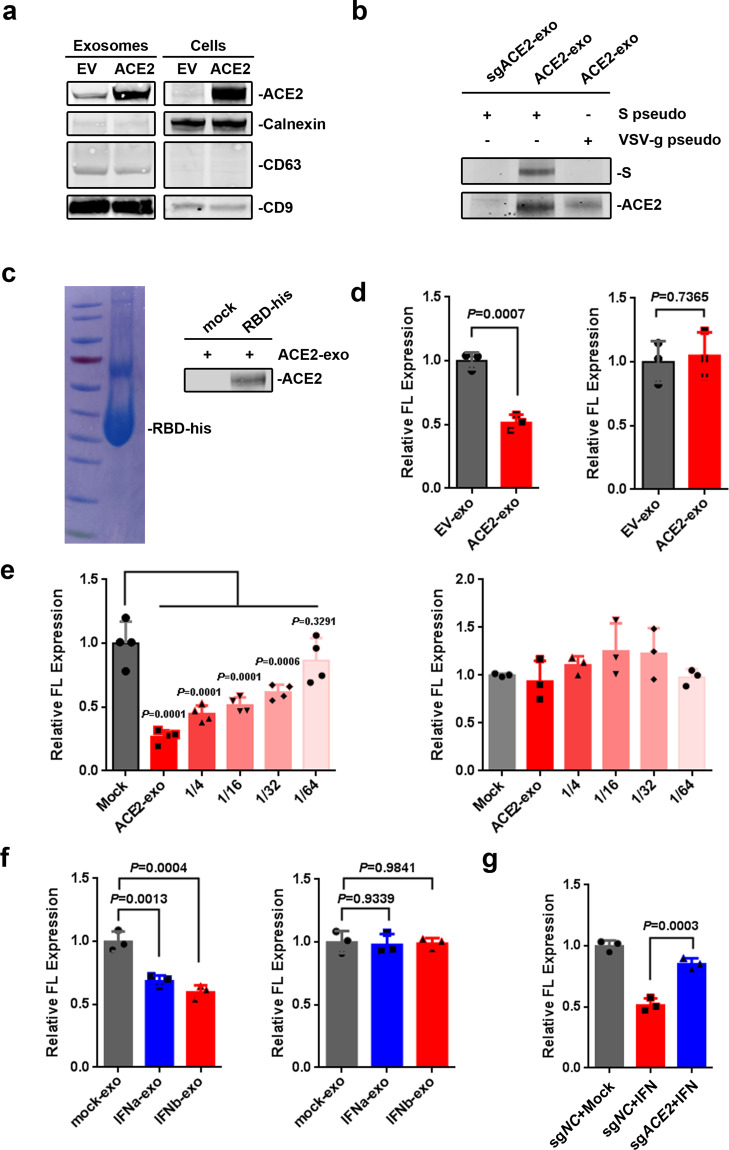
Exosomal hACE2 competitively blocks the cell entry of SARS-CoV-2. **a** Western blot analysis of the whole cell lysates and purified exosomes from HEK293T cells transfected with 5 μg of empty vector (EV) or hACE2-expresssing plasmid (ACE2). **b** The interaction between purified exosomes and pseudovirions. The purified exosomes derived from sg*ACE2* (sg*ACE2*-1) cell line (sg*ACE2*-exo) or HEK293T transfected with 1 μg of hACE2-expresssing plasmid (ACE2-exo) were incubated with purified SARS-CoV-2-S/HIV-1 pseudovirions (S pseudo) or VSV-g/HIV-1 pseudovirions (VSV-g pseudo), followed by enriched with Human CD63 Isolation/Detection Reagent and subsequently analyzed by western blot. **c** The interaction between purified RBD-his protein and purified exosomes. Left: Coomassie stain of purified RBD-his protein. Right: Purified exosomes from HEK293T cells transfected with 1 μg of hACE2-expresssing plasmid (ACE2-exo) were incubated with or without purified RBD-his protein, followed by enriched with a Ni-conjugated agarose bead column and subsequently analyzed by western blot. **d** Effect of purified exosomes on the entry of pseudovirions. SARS-CoV-2-S/HIV-1 pseudovirions (Left) or VSV-g/HIV-1 pseudovirions (Right) were mixed with purified exosomes derived from HEK293T cells transfected with 5 μg of EV (EV-exo) or hACE2-expresssing plasmid (ACE2-exo) for 5 min at room temperature, and added into HEK293T cells on 96-well plate. Cells were harvested for luciferase activity at 40 h post inoculation. **e** Exosomal hACE2 competitively the entry of SARS-CoV-2-S/HIV-1 pseudovirions. SARS-CoV-2-S/HIV-1 pseudovirions (Left) or VSV-g/HIV-1 pseudovirions (Right) were mixed with serial diluted concentration of ACE2-exo derived from HEK293T cells transfected with 15 μg hACE2-expresssing plasmid, and added into HEK293T cells on 96-well plate. Cells were harvested for luciferase activity at 40 h post inoculation. **f** Effect of purified exosomes on the entry of pseudovirions. SARS-CoV-2-S/HIV-1 pseudovirions (Left) or VSV-g/HIV-1 pseudovirions (Right) were mixed with purified exosomes derived from HEK293T cells treated with IFNα/β (50 ng/ml for 36 h) for 5 min at room temperature, and added into HEK293T cells on 96-well plate. Cells were harvested for luciferase activity at 40 h post inoculation. **g** Effect of purified exosomes derived from sg*NC* cell lines or sg*ACE2* cell lines in the presence of IFNα/β treatment on the entry of pseudovirions. SARS-CoV-2-S/HIV-1 pseudovirions were mixed with purified exosomes derived from sg*NC* cell lines or sg*ACE2* cell lines treated with IFN (25 ng/ml IFNα plus 25 ng/ml IFNβ for 36 h) for 5 min at room temperature, and added into HEK293T cells on 96-well plate. Cells were harvested for luciferase activity at 40 h post inoculation. Data were shown as the mean ± SD of three or four independent experiments, and compared using he two-tailed student’s *t* test (**d**) or one-way ANOVA test (**e–g**), in which each column compared to the first column of each graph. **P < 0.01; ***P < 0.001; ns not significant

ACE2 is the receptor for the cell entry of SARS-CoV-2, and cryoelectron microscopy experiments have recently demonstrated that the S protein of SARS-CoV-2 had high binding affinity to hACE2.^10–13^ Therefore, we wonder whether the hACE2 on the surface of exosomes could bind to the S protein on the surface of SARS-CoV-2-S/HIV-1 pseudovirions, and thus block the cell entry of the viruses. To increase the expression of hACE2 in exosomes, HEK293T cells were transfected with hACE2-expressing plasmid to produce excess hACE2-containing exosomes (ACE2-exo) (Fig. 3a and Supplementary Fig. 3a). After co-culture of the purified ACE2-exo with the enriched SARS-CoV-2-S/HIV-1 pseudovirions, the exosome-specific isolation Dynabeads (Exosome-Human CD63 Isolation/Detection Reagent) was used to purify exosomes. We found that exosomal hACE2 interacted with S protein on the surface of SARS-CoV-2-S/HIV-1 pseudovirions (Fig. 3b). Furthermore, the purified RBD of S protein also showed specific interaction with ACE2-exo (Fig. 3c). Of note, the purified RBD protein has competitive activity against SARS-CoV-2-S/HIV-1 pseudovirions infection but not against VSV-g/HIV-1 pseudovirions infection (Supplementary Fig. 3b, c), indicating that the purified RBD is of the biological activity. These data show that hACE2 on the surface of exosomes can specifically interact with RBD domain of S protein.

Based on the results, we speculated that ACE2-exo may be a powerful interfering tool for SARS-CoV-2 infection. Indeed, when we co-cultured ACE2-exo with pseudovirions, the cell entry of SARS-CoV-2-S/HIV-1 pseudovirions was significantly reduced compared with exosome isolated from cells transfected with empty vector (EV-exo), whereas the cell entry of VSV-g/HIV-1 pseudovirions was not affected (Fig. 3d). ACE2-exo inhibited the cell entry of SARS-CoV-2-S/HIV-1 pseudovirions in a dose-dependent manner, indicating that ACE2-exo can competitively block the entry of SARS-CoV-2-S/HIV-1 pseudovirions (Fig. 3e). Accordingly, exosomes isolated from cells treated with IFNα (IFNα-exo) or IFNβ (IFNβ-exo) also reduced the cell entry of SARS-CoV-2-S/HIV-1 pseudovirions but not VSV-g/HIV-1 pseudovirions since IFNα/β treatment stimulated the expression of exosomal hACE2 (Fig. 3f). Importantly, the inhibitory effect of IFNα/β-exo on cell entry of SARS-CoV-2-S/HIV-1 pseudovirions was diminished when exosomes derived from sg*ACE2* cell lines (Fig. 3g), indicating that hACE2 expression on the exosomes contributes to IFNα/β-exo-mediated antiviral effect. These results demonstrate that exosomal hACE2 inhibits the attachment of SARS-CoV-2 to the cells, and then competitively blocks the cell entry of the virus.

### Exosomal hACE2 inhibits SARS-CoV-2 infection in vitro and ex vivo

**Fig. 4:**
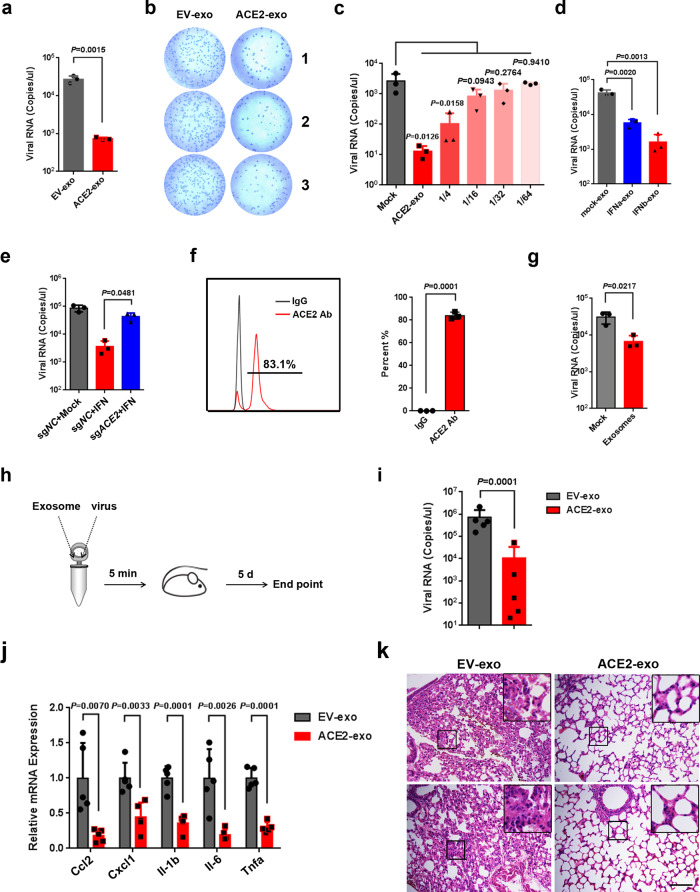
Exosomal hACE2 inhibits SARS-CoV-2 infection in vitro and ex vivo. **a** Inhibitory effect of ACE2-exo on the wild-type SARS-CoV-2 infection. Wild-type SARS-CoV-2 were mixed with purified exosomes derived from HEK293T cells transfected with 5 μg of EV (EV-exo) or hACE2-expresssing plasmid (ACE2-exo) for 5 min at room temperature, and infected Vero E6 cells on 48-well plate. Cells were harvested for viral titer at 48 h post infection. **b** Wild-type SARS-CoV-2 were mixed with purified exosomes derived from HEK293T cells transfected with 5 μg of EV (EV-exo) or hACE2-expresssing plasmid (ACE2-exo) for 5 min at room temperature, and infected Vero E6 cells on 96-well plate, followed by plaque reduction assays at 24 h post infection. **c** Exosomal hACE2 competitively inhibits wild-type SARS-CoV-2 infection in vitro. Wild-type SARS-CoV-2 were mixed with serial diluted concentration of ACE2-exo derived from HEK293T cells transfected with 15 μg hACE2-expresssing plasmid, and infected Vero E6 cells on 96-well plate. Cells were harvested for viral titer at 48 h post infection. **d** Wild-type SARS-CoV-2 were mixed with purified exosomes derived from HEK293T cells treated with IFNα/β (50 ng/ml for 36 h) for 5 min at room temperature, and infected Vero E6 cells on 48-well plate. Cells were harvested for viral titer at 48 h post infection. **e** Wild-type SARS-CoV-2 were mixed with purified exosomes derived from sg*NC* cell lines or sg*ACE2* cell lines treated with IFN (25 ng/ml IFNα plus 25 ng/ml IFNβ for 36 h) for 5 min at room temperature, and infected Vero E6 cells on 48-well plate. Cells were harvested for viral titer at 48 h post infection. **f** The purified exosomes derived from HEK293T cells infected with SARS-CoV-2 were further enriched with Human CD63 Isolation/Detection Reagent and stained with anti-ACE2 antibody, followed by flow cytometry analysis (Left panel). Right: Frequency of ACE2^+^ exosomes in left. **g** The exosomes from HEK293T cells infected with SARS-CoV-2 (MOI = 0.001) were purified by differential ultracentrifugation, and further enriched with Human CD63 Isolation/Detection Reagent. Wild-type SARS-CoV-2 were mixed with the enriched exosomes or not for 5 min at room temperature, and infected Vero E6 cells on 48-well plate. Cells were harvested for viral titer at 48 h post infection. Data were shown as the mean ± SD of three independent experiments, and compared using he two-tailed student’s *t* test (**a**, **f**, **g**) or one-way ANOVA test (**c–e**), in which each column compared to the first column of each graph. *P < 0.05; **P < 0.01; ns not significant. **h** Schematic for the ex vivo experiment. hACE2-mice (*n* = 5 each group) were challenged i.n. with wild-type SARS-CoV-2 pre-inoculation with purified exosomes derived from HEK293T cells transfected with 5 μg of EV (EV-exo) or hACE2-expresssing plasmid (ACE2-exo) for 5 min at room temperature. Mice were sacrificed at day 5 post infection, and lungs were collected for viral titers (**e**), analysis of inflammatory factors (**j**), or histopathological analysis (**k**). Scale bar, 100 μM. Data were shown as the mean ± SD, and compared using the two-tailed student’s *t* test (**i**) or multiple *t* tests (**j**). **P < 0.01; ***P < 0.001

To further study, the therapeutic potential of ACE2-exo, Vero E6 cells were infected with wild-type SARS-CoV-2 at a MOI of 0.02, which was pre-treated with EV-exo or ACE2-exo. SARS-CoV-2 infection was significantly inhibited after co-culture with ACE2-exo compared to EV-exo (Fig. 4a, b). ACE2-exo also inhibited the SARS-CoV-2 replication in a dose-dependent manner (Fig. 4c). Similarly, SARS-CoV-2 infection was also reduced after co-incubated with IFNα/β-exo (Fig. 4d). However, the inhibitory effect of IFNα/β-exo on SARS-CoV-2 replication diminished when derived from sg*ACE2* cell lines (Fig. 4e), further indicating that hACE2 expression on the exosomes contributes to IFNα/β-exo-mediated anti-SARS-CoV-2 effect. To analyze whether hACE2 is expressed on the exosomes derived from SARS-CoV-2-infected cells, we purified exosomes from SARS-CoV-2-infected cells found that hACE2-containing exosomes were secreted from the infected cells (Fig. 4f). To further explore whether exosomal hACE2 secreted from the infected cells could interfere with virus infection, we used low MOI of SARS-CoV-2 (MOI = 0.001) to allow more free exosomal hACE2 exist on the exosomes. In the meantime, to separate exosomes from viruses, purified exosomes were further enriched with Exosome-Human CD63 Isolation/Detection Reagent. The enriched exosomes were utilized to block the SARS-CoV-2. The result showed that SARS-CoV-2 infection was inhibited after co-culture with the enriched exosomes when compared to the negative control (culture medium) (Fig. 4g), indicating that hACE2-containing exosomes were secreted from the infected cells and could interfere with virus infection. These data show that exosomal hACE2 inhibits SARS-CoV-2 infection in vitro.

Human ACE2 transgenic mice (hACE2-mice) are highly susceptible to SARS-CoV-2 infection.^42^ To further test the antiviral efficiency of ACE2-exo ex vivo, we utilized these hACE2 transgenic mice in our study. SARS-CoV-2 were pre-treated with EV-exo or ACE2-exo, and then infected hACE2 transgenic mice with 1× 10^5^ PFU intranasally (i.n.). All mice were sacrificed at day 5 post infection (dpi) for viral load and inflammatory factors analysis (Fig. 4h). As expected, mice infected with viruses pre-treated with ACE2-exo showed lower viral load in lung (Fig. 4i). SARS-CoV-2 infection can cause fatal inflammatory responses and acute lung injury.^42,43^ A decrease in the mRNA expression of pro-inflammatory cytokines/chemokines and inflammation in lung was observed in the mice infected with viruses pre-treated with ACE2-exo (Fig. 4j, k). Taken together, exosomal hACE2 reduces SARS-CoV-2 infection in vitro and ex vivo, and ACE2-exo may be taken as a therapeutic intervention for COVID-19 therapy.

## Discussion

hACE2 protein is a type I integral membrane glycoprotein containing a single catalytic domain and degrades Angiotensin (Ang) II, and thus plays as a negative regulator in the renin-angiotensin system (RAS).^44,45^ It is ubiquitous expressed in the heart, vessels, gut, lung, liver, intestine, kidney, testis, and brain,^45^ and is a negative regulator in the RAS and degrades Ang II to Ang (1–7). The resulting Ang (1–7) will trigger vasodilation, anti-inflammatory, anti-fibrotic, and anti-proliferative effects.^46^ As ACE2 is a potent regulator in the pathological changes in several organs, including lungs, heart, testes, and kidneys, its expression is closely related to the clinical symptoms of COVID-19.^47,48^ Since the S protein of SARS-CoV-2 binds to hACE2 through membrane fusion, the infection of SARS-CoV-2 may cause systematic deprivation of ACE2,^49^ which could result in increased inflammation, vasoconstriction, and thrombosis.^50^ Severe COVID-19 patients like hypertension, diabetes, cardiovascular disease share a degree of ACE2 deficiency suggest the pivotal role of hACE2 in COVID-19.^51,52^ hrsACE2 was reported to bind to S protein of SARS-CoV-2 and block the replication of SARS-CoV-2.^16^ Our data further show that the exosomal hACE2 significantly blocks the entry of SARS-CoV-2 and reduces SARS-CoV-2 replication in vitro and ex vivo, providing an alternative tool for the treatment of SARS-CoV-2 infection. Inhibition of SARS-CoV-2 infection with exosomal hACE2 may be a promising treatment since it can specifically inhibit SARS-CoV-2 without reducing the level of hACE2.

Exosomes have been utilized as a potential therapeutic tool including the natural exosomes, exosomes with modifications, and exosomes loaded with exogenous cargo.^53^ Naturally secreting exosomes derived from lung spheroid cells (LSC-Exo) have recently been reported to promote lung repair in pulmonary fibrosis through inhalation.^54^ As ACE2 is a key regulator of lung pathology and protect the lung from injury, our results may partially explain the mechanism that LSC-Exo promotes the lung repair. However, more experiments are needed to support this hypothesis. Based on our results, it may be more sufficient tools when exosomes are modified or loaded with cargos, such as exosomes modified with increasing ACE2 expression on exosomes or exosomes loaded with exogenous anti-SARS-CoV-2 drugs. Also, inhalation of LSC-Exo with ACE2 upregulation may be a safer and more efficient way for the treatment of SARS-CoV-2 infection since SARS-CoV-2 mainly entry into host through airway system. However, our data only provided an ex vivo data that ACE2-exo could inhibit the replication of SARS-CoV-2. More experiments especially experiments testing the efficiency and safety of inhalation of ACE2-exo in vivo are needed in the future.

During the cell entry of SARS-CoV-2, the viruses take advantage of the host endocytosis pathway.^10^ The maturation of endosomes is also involved in endocytosis pathway.^25^ It was reported that mice injected with SARS-CoV spike showed a decrease in the ACE2 expression, and thereby resulted in lung injury.^15,55^ In addition to that the fusion of SARS-CoV S with membrane receptor ACE2 induces a decrease in ACE2, we speculated that the S protein of SARS-CoV-2 may interfere with the production of exosomal hACE2 and decrease the level of exosomal hACE2. However, more evidences are needed to prove the hypothesis. Importantly, since the cell entry of SARS-CoV-2 and the production of exosomal hACE2 both rely on endocytosis, more careful and well-controlled experimentations or clinical trials are required when treated COVID-19 with the inhibitors of endocytosis.

Type I IFNs have been approved to use in the treatment of many diseases including viral infections (hepatitis B and hepatitis C), autoimmune diseases and certain cancers, and have currently been used in clinical trials to treat MERS-CoV and SARS-CoV-2.^37–39^ hACE2 is currently identified as an IGS and induced by both type I and type III IFNs in primary human upper airway basal cells.^40^ Accordingly, our data also showed that hACE2 was stimulated by type I IFNs in human airway cell lines and human embryonic kidney cells, highlighting that SARS-CoV-2 could utilize ISG hACE2 as receptor to challenge host defense systems. However, we found that hACE2 is expressed on exosomes and upregulated by type I IFNs, and exosomal hACE2 inhibited SARS-CoV-2 infection. To avoid the evasion by SARS-CoV-2, hACE2 exosomes or IFN-induced exosomes seem to be a more efficient tool for the treatment of SARS-CoV-2 infection. Our data have also presented a novel antiviral mechanism that a viral receptor, which is an ISG product and expressed on the exosomes, potently blocks the virus entry.

## Materials and methods

### Cells culture and transfection

Human embryonic kidney 293T (HEK293T) cells, Vero E6 and human lung cancer H1299 cells were obtained from American Type Culture Collection and cultured in Dulbecco’s modified Eagle’s medium (ThermoFisher Scientific) supplemented with 10% fetal bovine serum (FBS, ThermoFisher Scientific) or exosome-depleted FBS (SBI), 100 units/ml penicillin (ThermoFisher Scientific), and 100 μg/ml streptomycin (ThermoFisher Scientific) at 37 °C with 5% CO_2_. 16HBE cells were maintained in Roswell Park Memorial Institute 1640 medium (ThermoFisher Scientific) supplemented with 10% FBS or exosome-depleted FBS, and 100 units/ml penicillin, and 100 μg/ml streptomycin at 37 °C with 5% CO_2_. HEK293F cells were maintained in Expi293™ Expression Medium (ThermoFisher Scientific) supplemented with 100 units/ml penicillin, and 100 μg/ml streptomycin at 37 °C with 5% CO_2_. HEK293T cells were transfected with the indicated plasmids using Lipofectamine 2000 (ThermoFisher Scientific) according to the manufacturer’s instructions.

### Plasmids and constructs

Plasmid expressing Flag-tagged human ACE2 cDNA ORF was purchased from Sino Biological lnc. The plasmid containing codon-optimized cDNA of the spike (S) gene of SARS-CoV-2 was purchased from Generay Biotech company (Shanghai, China) and cloned into the pcDNA3.1 vector. The plasmid containing 6×his-tagged RBD of S protein were cloned into pcDNA3.1 vector containing IRES-GFP-WPRE element. IFNα2β was purchased from Sino Biological lnc and IFNβ1 were purchased from Peprotech. Primary antibodies used in the study include anti-ACE2 rabbit polyclonal antibody (Sino Biological lnc), anti-Calnexin rabbit polyclonal antibody (Proteintech), anti-GAPDH rabbit polyclonal antibody (Proteintech), anti-CD63 rabbit polyclonal antibody (Cell Signaling Technology, CST), anti-CD9 mouse monoclonal antibody (Proteintech), anti-Hrs rabbit polyclonal antibody (Proteintech), PE-anti-CD63 Monoclonal Antibody (ThermoFisher Scientific), anti-SARS-N polyclonal antibody (Sino Biological lnc), and anti-S1 rabbit polyclonal antibody (Sino Biological lnc).

### Production of pseudovirions and virus entry

SARS-CoV-2-S/HIV-1 pseudovirions or VSV-g/HIV-1 pseudovirions were produced from cells co-transfected with SARS-CoV-2-S expressing plasmid or VSV-G expressing plasmid together with pHIV-luciferase, psPAX2. The supernatants were collected at 48 h post transfection, filtered through a 0.45 μm pore-size filter and stored at −80 °C for used. To test the cell entry of pseudovirions, cells were seeded on 96-well plates and inoculated with 100 μl media containing pseudovirions. The cells were harvested at 40 h post inoculation and lysed with 100 μl 1× Steady-glo (Promega). The luciferase activity was analyzed with Luciferase Reporter Assay Kit (Promega) according to the manufacturer’s instructions.

### Inhibition of SARS-CoV-2 replication

The experiment of inhibition of wild-type SARS-CoV-2 named as hCoV-19/CHN/SYSU-IHV/2020 strain (Accession ID on GISAID: EPI_ISL_444969) was performed in a biosafety level 3 (BSL3) facility at Sun Yat-sen University (SYSU). The inhibitory effect of purified exosomes on SARS-CoV-2 replication was determined by qRT-PCR and plaque reduction assays. The supernatants from cells infected with SARS-CoV-2 co-cultured with exosomes derived from HEK293T cells transfected with empty vector (EV-exo), exosomes derived from HEK293T cells transfected with hACE2-expresssing plasmid (ACE2-exo), or diluted ACE2-exo were harvested at 48 h post infection. The viral RNA copy was determined by qRT-PCR using virus detection Kit from DA AN GENE CO., LTD OF SUN YAT-SEN UNIVERSITY.

For testing the inhibitory effect of purified exosomes SARS-CoV-2 replication with plaque reduction assays,^56^ Vero E6 cells plated on a 96-well plate were infected with SARS-CoV-2 pre-inoculated with EV-exo or ACE2-exo at 37 °C for 1 h. The supernatant was removed, and 1.6% warmed CMC was overlaid on the cells. The plate was fixed with 4% paraformaldehyde (PFA) and stained with anti-SARS-N polyclonal antibody at day 1 post infection, followed by stained with secondary antibody. The plate was developed with TrueBlue (KPL) at room temperature for 5–10 min, and counted using an EliSpot reader (Cellular Technology Limited, CTL).

### Purification and verification of exosomes

Cells were cultured in media supplemented with 10% exosome-depleted FBS. Supernatants from cells cultured for 48–72 h were collected exosomes were purified with a standard differential ultracentrifugation,^34,57,58^ at 300 g for 10 min, 2000 g for 10 min, 10,000 g for 30 min, and 140,000 g for 70 min, followed by one wash with PBS and centrifugation at 140,000 g for 70 min.

To identify the purified exosomes with TEM, purified exosomes were suspended in PBS and dropped onto a copper mesh and settled for 1 min. Filter paper was used to absorb the floating liquid. The copper mesh was added with 2% phosphotungstic acid for 1 min, followed by absorbed the floating liquid with filter paper. After the mesh was dried for several minutes, the morphology of the exosomes was analyzed with HT7700 transmission electron microscope (Hitachi). For immunogold labeling, purified exosomes derived from HEK293T cells suspended with 2% PFA were placed on formvar carbon-coated nickel grids, blocked, and incubated with anti-ACE2 primary antibody (1:50 dilution, Sino Biological lnc), followed by incubation with the anti-rabbit secondary antibody conjugated with protein A-gold particles (10 nm). To verify the size and concentration of exosomes with NTA, purified exosomes were suspended in PBS and analyzed using a ZetaVIEW S/N 17-310 (PARTICLE METRIX). For analysis of purified exosomes with flow cytometry, purified exosomes were further enriched with Exosome-Human CD63 Isolation/Detection Reagent (from cell culture media) (ThermoFisher Scientific), and stained with primary antibodies according to the manufacturer’s instructions, followed by flow cytometry analysis LSRFortessa flow cytometer (BD Bioscience). Data were analyzed with the FlowJo V10.0.7 (FlowJo).

### Generation of stable hACE2 or Hrs knockdown cell lines

Oligonucleotides of sgRNAs against human ACE2 (sg*ACE2*-1: ACTTTGATAGAACAGGTCTT, sg*ACE2*-2: TCAGTCCACCATTGAGGAAC) were inserted into LentiCRISPRv2 vector (Addgene). Short hairpin RNAs (shRNAs) against human Hrs (also known as HGS) (sh*Hrs*-1: GCACGTCTTTCCAGAATTCAA, sh*Hrs*-2: GCATGAAGAGTAACCACAGC),^29^ or scrambled shRNA-control were designed and cloned into pLKO.3G vector according to the previous study.^59^ The resulting lentiviral plasmids were transfected into HEK293T cells together with psPAX2 and VSV-g. The supernatants were collected at 48 h post transfection, and filtered through a 0.45 μm pore-size filter. Cells were infected with the lentiviruses. Positive clone were selected by 2 μg/ml puromycin (sgRNAs) or selected by flow cytometry (shRNAs) at 48 h post infection.

### Western blot assay

Western blot assay was performed as described previously.^59–61^ Supernatants derived from ~2–4 plates of HEK293T (15 cm plate) or derived from eight plates of 16HBE or H1299 cells were collected for purified exosomes. Cells or purified exosomes were lysed and the concentration was measured by the Bradford method. The lysates were separated by SDS-PAGE and transferred onto nitrocellulose membranes. The membranes were further blocked with 5% non-fat dry milk at room temperature for 1 h, and incubated with primary antibodies at room temperature for 1 h, followed by stained with secondary antibodies at room temperature for 1 h. CD9 and CD63 were used as exosome markers, while Calnexin and GAPDH were used as whole cell lysates loading control.

### ELISA

The expression of exosomal hACE2 was tested with QuantiCyto^®^ Human ACE2 ELISA kit (Neobioscience) according to the manufacturer’s instructions. The purified exosomes were added into ELISA plates coated with monoclonal antibody against hACE2 at 37 °C for 1.5 h, followed by washing with 1× washing buffer for five times. Biotinylated monoclonal hACE2 antibody was then added to each well and incubated for 1 h at room temperature, followed by washing with 1× washing buffer for five times. Subsequently, the diluted horseradish peroxidase-conjugated streptavidin was added to each well and incubated for 30 min at room temperature, followed by washing with 1× washing buffer for five times. The plates were developed with TMB buffer for 15 min, and stopped with 1× stop buffer. The plates were read at 450 nm with a MK3 plate reader (ThermoFisher scientific).

### Immunofluorescence assay (IFAs)

IFAs was performed as previously described.^59,62^ In brief, HEK293T cells were seeded on in μ-slide chambered coverslips (Ibidi; 80826). Cells were fixed with 4% poly-formaldehyde in room temperature for 10 min, then permeabilized with 1% triton 100 in PBS for 15 min and blocked with 5% BSA PBS for 30 min. Cells were incubated with primary antibodies at room temperature for 1 h. After washing with 0.1% Tween-20 PBS for three times, cells were stained with secondary antibodies for 1 h, and 4′,6-diamidino-2-phenylindole dihydrochloride for 5 min. Images were obtained with LSM880 confocal microscopy (Zeiss).

### Exosomes and pseudovirions binding assay

To pellet down pseudovirions, the viral supernatants were centrifuged at 28,000 rpm in a Beckman SWi32 rotor at 4 °C for 2 h. The viral pellets were resuspended with 0.1% BSA PBS. The resuspended pseudovirions were incubated with purified exosomes at 4 °C for 2 h, followed by enrichment using human CD63 isolation/Detectoion according to the manufacturer’s instructions. The exosome-bound pseudovirions were detected by western blot.

### Purification of RBD of S protein and its interaction with exosomes

The plasmid harboring 6×His-tagged RBD was transfected into HEK293F cells using polyetherimide. Supernatants were collected at 48 h p.t. and filtered through a 0.45 μm pore-size filter. The supernatants were subsequently applied to a Ni-conjugated agarose bead column (GE Healthcare, Buckinghamshire, UK). After washing with 30 mM Imidazole (Sangon Biotech, Shanghai, China), the bound His fusion proteins were eluted with 500 mM Imidazole. Finally, the proteins were suspended in PBS buffer and the concentration was measured by the Bradford method. The purified RBD was incubated with purified exosomes at 4 °C for 4 h, followed by enriched with a Ni-conjugated agarose bead column. The bound protein was further washed with PBS buffer containing 0.25% NP40 and 50 mM NaCl for three times and eluted in protein gel loading buffer. The eluted samples were then analyzed by SDS-PAGE and detected by western blot assay.

### Mice and ex vivo testing assay

Human ACE2 transgenic mice (hACE2-mice) (Gempharmatech) were utilized in the study. All animal experiments were approved by Ethics Committee of Zhongshan School of Medicine (ZSSOM) on Laboratory Animal Care and were carried out in strict accordance with guidelines and regulations of Laboratory Animal Center of ZSSOM, SYSU, Guangzhou (China). Experiments involved mice infected with wild-type SARS-CoV-2 were performed in an animal BSL3 lab at SYSU. hACE2-mice at 6-week-old were infected i.n. with wild-type SARS-CoV-2 pre-treated with EV-exo or ACE2-exo, respectively. Mice were sacrificed at day 5 post infection. The lungs of the mice were removed for determination of viral titers by qRT-PCR using virus detection Kit from DA AN GENE CO., LTD OF SUN YAT-SEN UNIVERSITY, for the analysis of inflammatory factors using qRT-PCR, and for the testing of lung pathology using histopathological analysis.

### Quantitative PCR (qPCR)

Total RNA isolation and qRT-PCR were performed as previously described.^62,63^ The specific primers for the target genes were also used as previously described.^63^

### Histopathological analysis

Lungs of each mouse were fixed in formalin, embedded in paraffin, and stained with haematoxylin and eosin as previously described.^63^

### Statistical analysis

Data were analyzed using GraphPad 6.0 software (La Jolla, CA, USA). The two-tailed Student’s *t* test, one-way ANOVA, and multiple *t* tests were used to determine the significance of statistical data. Experiments were repeated twice, and data were shown as the mean ± SD of three or four independent experiments, and considered significant at *P < 0.05, **P < 0.01, and ***P < 0.001.

## Supplementary information

Supplementary figures

## Data Availability

The data used and/or analyzed to support the findings of this study are available in this paper or the Supplementary Information. Any other raw data that support the findings of this study are available from the corresponding author upon reasonable request.
