# Supplementary material for: The interferon-stimulated exosomal hACE2 potently inhibits SARS-CoV-2 replication through competitively blocking the virus entry
Source: Signal Transduct Target Ther. 2021 May 12;6:189. doi: 10.1038/s41392-021-00604-5 (PMC8113286; doi:10.1038/s41392-021-00604-5)
Supplement: Supplementary file 1 — Supplementary figures [file 41392_2021_604_MOESM1_ESM.docx]

Supplementary Materials for

The Interferon-stimulated Exosomal hACE2 Potently Inhibits SARS-CoV-2 Replication through Competitively Blocking the Virus Entry

Junsong Zhang, Feng Huang, Baijin Xia, Yaochang Yuan, Fei Yu, Guanwen Wang, Qianyu Chen, Qian Wang, Yuzhuang Li, Rong Li, Zheng Song, Ting Pan, Jingliang Chen, Gen Lu, Hui Zhang

Correspondence to: zhangh92@mail.sysu.edu.cn

**This PDF file includes:**

Figures. S1 to S3


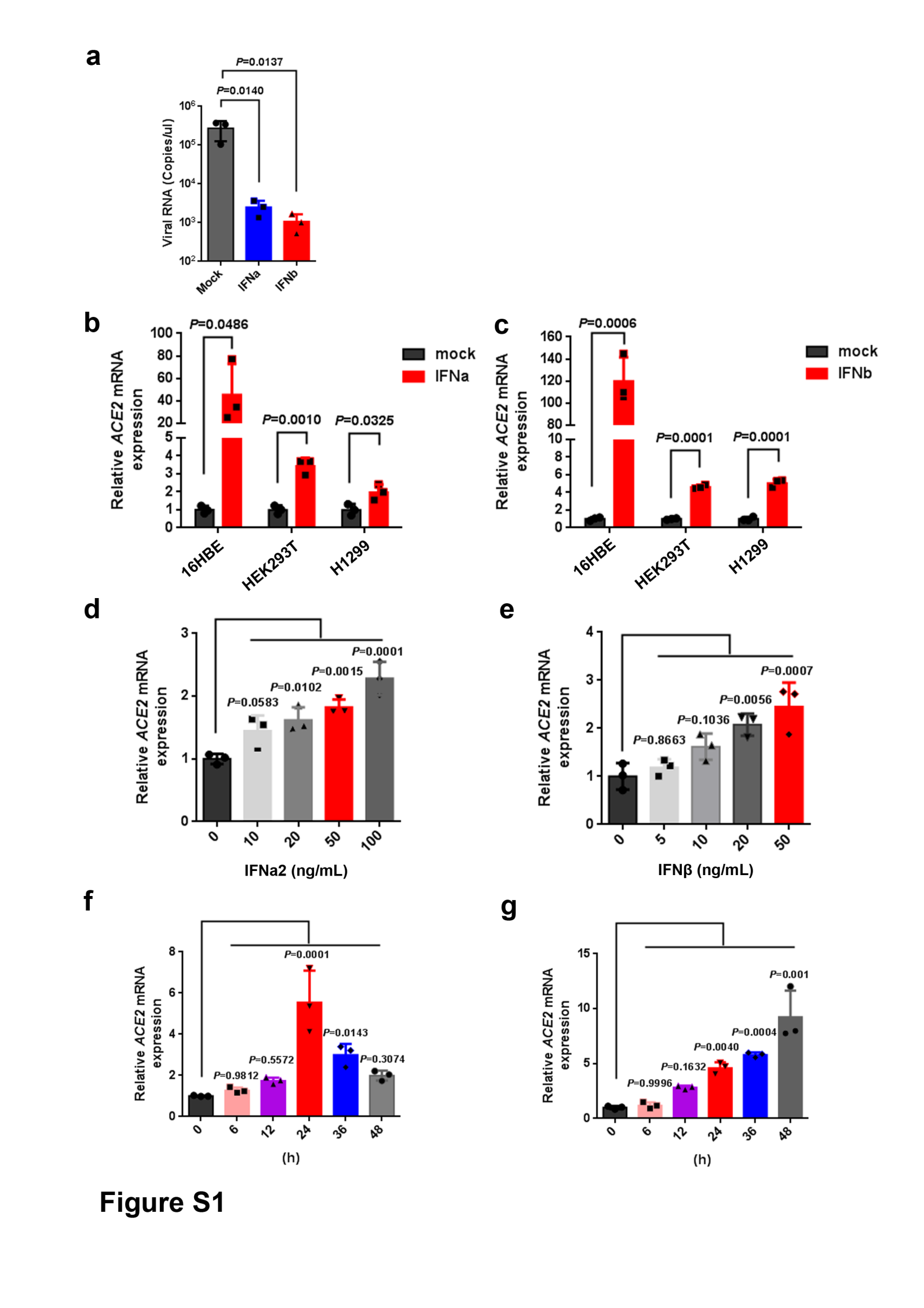


Figure. S1.

Human ACE2 is regulated by type I IFN in different cell lines. a, The inhibitory effect of IFNα/β on the replication of wild-type SARS-CoV-2. Vero E6 cells were treated with 50 ng/ml IFNα, IFNβ or not for 12 h, followed by infected with wild-type SARS-CoV-2 (MOI=0.02) for 1h. Cells were harvested for viral titer at 48 h post infection. b, The expression of intracellular hACE2 from different cell lines treated with IFNα (50 ng/ml for 12 h) or not (mock) was analyzed by qRT-PCR. c, The expression of intracellular hACE2 from different cell lines treated with IFNβ (50 ng/ml for 12 h) or not (mock) was analyzed by qRT-PCR. d, The expression of intracellular hACE2 from HEK293T cells treated with different dose of IFNα for 12 h was analyzed by qRT-PCR. e, The expression of intracellular hACE2 from HEK293T cells treated with different dose of IFNβ for 12 h was analyzed by qRT-PCR. f, HEK293T cells were treated with 50 ng/mL IFNα and collected at different time points. The expression of intracellular hACE2 was then analyzed by qRT-PCR. g, HEK293T cells were treated with 50 ng/mL IFNβ and collected at different time points. The expression of intracellular hACE2 was then analyzed by qRT-PCR. Data were shown as the mean ± SD of three independent experiments, and compared using student t test (a), the one-way ANOVA test (d-g) or multiple t tests (b and c). *, *P*<0.05; **, *P*<0.01; ***, *P*<0.001.


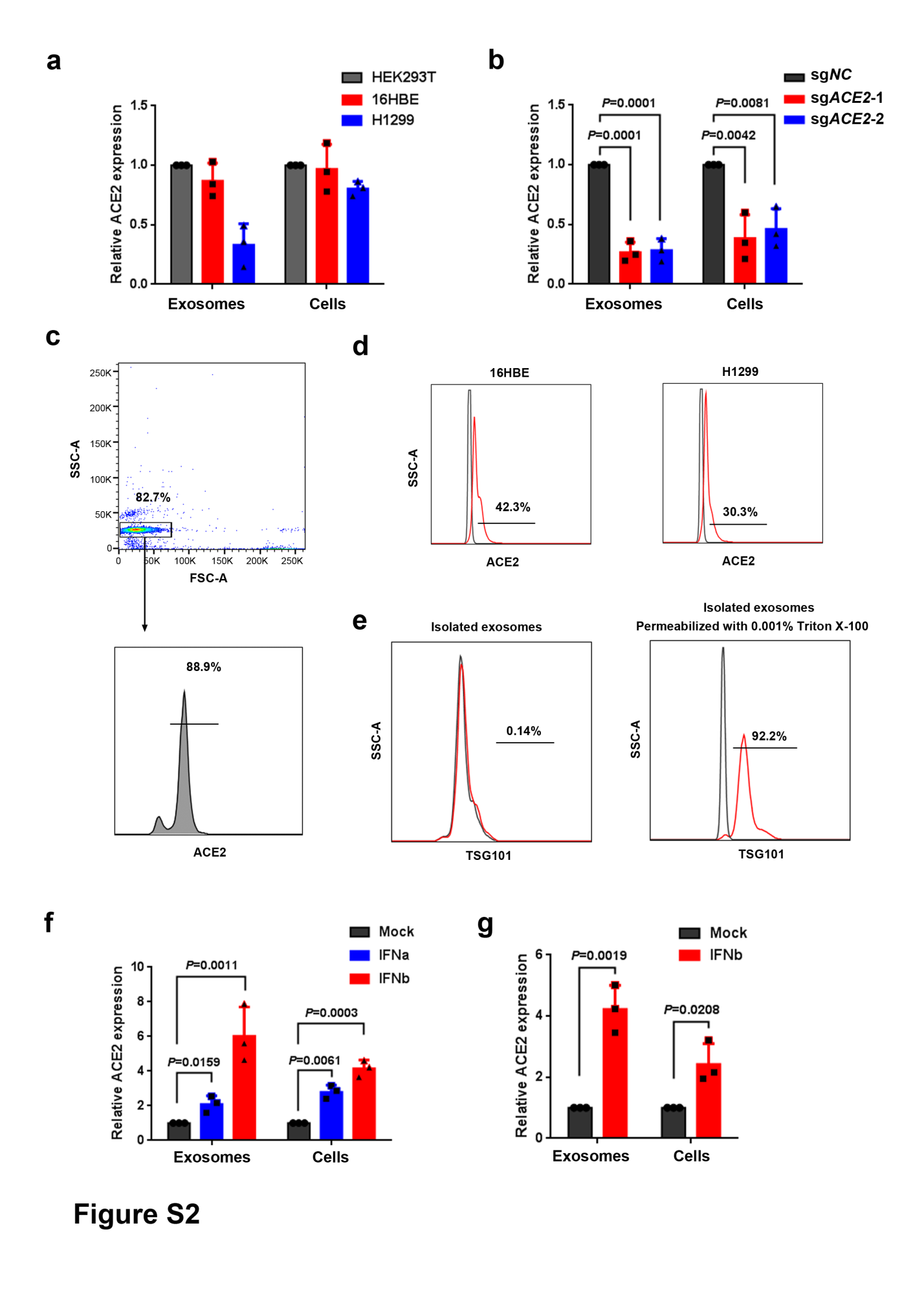


Figure. S2.

**hACE2 is specifically expressed on the surface of exosomes derived from different cell lines. a**, Statistical analysis of ACE2 levels in Figure 1d. The value for exosomal ACE2 was standardized to exosomal CD9 levels and normalized to the level of exosomal ACE2 derived from HEK293T cells, while the value for intracellular ACE2 was standardized to intracellular calnexin and normalized to the level of intracellular ACE2 in HEK293T cells. **b**, Statistical analysis of ACE2 levels in Figure 1g. The value for exosomal ACE2 was standardized to exosomal CD9 levels and normalized to the level of exosomal ACE2 derived from sg*NC* cell line, while the value for intracellular ACE2 was standardized to intracellular calnexin and normalized to the level of intracellular ACE2 in sg*NC* cell line. **c**, Gating strategy for FACS analysis of exosomal hACE2 used in the study. **d,** The purified exosomes derived from 16HBE or H1299 cells were enriched with Human CD63 Isolation/Detection Reagent and stained with anti-ACE2 antibody, followed by flow cytometry analysis. **e**, Flow cytometry analysis of TSG101 within exosomes. The purified exosomes derived from HEK293T cells were enriched with Human CD63 Isolation/Detection Reagent and permeabilized with or without 0.001% Triton X-100, followed by staining with anti-TSG101 antibody and flow cytometry analysis. **f**, Statistical analysis of ACE2 levels in Figure 2A. The value for exosomal ACE2 was standardized to exosomal CD9 levels and normalized to the level of exosomal ACE2 derived from untreated cells (Mock), while the value for intracellular ACE2 was standardized to intracellular calnexin and normalized to the level of exosomal ACE2 in untreated cells (Mock). **g**, Statistical analysis of ACE2 levels in Figure 2c. The value for exosomal ACE2 was standardized to exosomal CD9 levels and normalized to the level of exosomal ACE2 derived from untreated cells (Mock), while the value for intracellular ACE2 was standardized to intracellular calnexin and normalized to the level of exosomal ACE2 in untreated cells (Mock). Data were shown as the mean ± SD of three independent experiments, and compared using multiple *t* test (a, b, f, and g). *, *P*<0.05; **, *P*<0.01; ***, *P*<0.001.


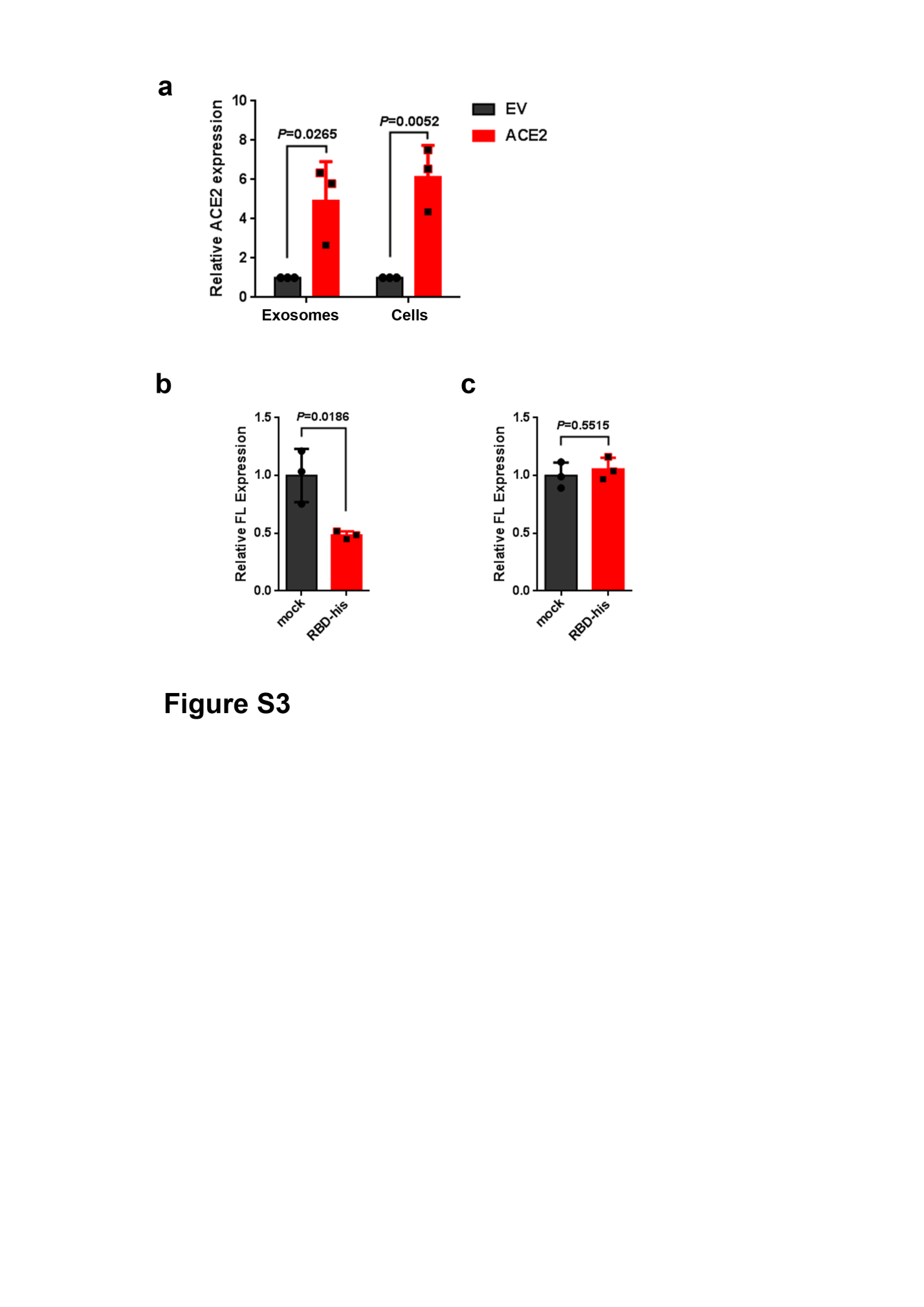


Figure. S3.

**The purified RBD protein can competitively block against the cell entry of SARS-CoV-2 S.**

**a**, Statistical analysis of ACE2 levels in Figure 3a. The value for exosomal ACE2 was standardized to exosomal CD9 levels and normalized to the level of exosomal ACE2 derived from cells transfected with empty vector (EV), while the value for intracellular ACE2 was standardized to intracellular calnexin and normalized to the level of exosomal ACE2 derived in cells transfected with empty vector (EV). **b** and **c**, SARS-CoV-2-S/HIV-1 pseudovirions (**b**) or VSV-g/HIV-1 pseudovirions (**c**) were mixed with 200 nM purified RBD protein for 5 min at room temperature, and added into HEK293T cells on 96-well plate. Cells were harvested for luciferase activity at 40 h post inoculation. Data were shown as the mean ± SD of three independent experiments using student *t* tests (b and c) or multiple *t* test (a). *, *P*<0.05; **, *P*<0.01; ***, *P*<0.001. ns: not significant.
